# Visual and ocular surface benefits of mini-scleral contact lenses in patients with chronic ocular graft-versus-host disease (GvHD)

**DOI:** 10.1038/s41598-024-76249-5

**Published:** 2024-10-24

**Authors:** Philip Keye, Susanne Issleib, Yvonne Gier, Mateusz Glegola, Philip Maier, Daniel Böhringer, Philipp Eberwein, Thomas Reinhard

**Affiliations:** 1 Eye Center, Medical Center - University of Freiburg, Faculty of Medicine, University of Freiburg, Freiburg, Germany; 2 Augenarztpraxis Prof. Grüb, Bahnhofstraße 7, 79206 Breisach, Germany; 3 Hecht Kontaktlinsen GmbH, Au, Germany; 4 Augencentrum Rosenheim, Rosenheim, Germany

**Keywords:** Outcomes research, Corneal diseases, Eye manifestations

## Abstract

Graft-versus-Host disease is a major complication of allogeneic stem cell transplantation. The eyes are a frequently affected organ with a severe dry eye disease being the hallmark manifestation. This retrospective study evaluates the effect of mini-scleral contact lenses on visual acuity, eye-related quality of life and the ocular surface. 62 eyes of 31 patients were included and visual acuity, ocular surface disease index (OSDI) questionnaire results and Oxford grades before and after mini-scleral lens fitting were compared. Median Snellen fraction with mini-scleral lenses was 20/25 (1st 20/30/3rd 20/20) compared to 20/40 median Snellen fraction with spectacles (1st 20/60/3rd 20/25). Median OSDI scores improved from 73 (1st 41.6/3rd 89) before fitting to 27 (1st 14.5/3rd 56) with mini-scleral lenses. Median Oxford grade decreased from 3 before mini-scleral lens fitting (1st 1/3rd 4.75) to 1 after mini-scleral lens fitting (1st 0/3rd 4). Median time of follow up was 717.5 days. Seven patients (22.6%) discontinued therapy with mini-scleral lenses. Mini-scleral lenses are beneficial for most patients with ocular GvHD as they improve visual acuity, eye-related quality of life and the integrity of the corneal epithelium.

## Introduction

Ocular graft-versus-host disease (GvHD) is a frequent complication of allogeneic hematopoietic stem cell transplantation. The hallmark of chronic ocular GvHD is severe dry eye disease^1,2^. Alloimmune inflammation and subsequent fibrosis of the lacrimal glands and the Meibomian glands leads to insufficient tear production and tear film instability with increased evaporation. Cicatricial conjunctival inflammation may lead to tarsal scarring with symblephara and severe ocular surface damage^2^. This exposes the cornea to an increased risk for microbial infections and ulceration. Both complications can permanently impair visual acuity.

Confirming the diagnosis of chronic ocular GvHD can be difficult as not all dry eye disease must necessarily occur due to ocular GvHD. Diagnostic measures include slit lamp microscopy with inspection of the eye lids, the conjunctiva and the cornea. Fluorescein staining of the ocular surface is used to evaluate epithelial damage and tear breakup time. Schirmer’s test is incorporated to quantify aqueous tear production. Further diagnostic tests such as meibography or measuring tear osmolarity are sometimes performed but not required for diagnosis. According to the NIH Consensus Development Project on Criteria for Clinical Trials in Graft-versus-Host Disease, clinical presentation with newly experienced symptoms of ocular discomfort (e.g. “gritty” eyes, painful conjunctivitis, photophobia, punctate keratitis) in combination with a reduced Schirmer’s test, are sufficient to establish the diagnosis for example in clinical trials conducted by non-ophthalmologists^3^. A more detailed diagnostic scheme and grading system was proposed by the International Chronic ocular graft-versus-host disease (GvHD) Consensus Group^4^.

Conventional treatment of ocular GvHD consists of lubrication with non-preserved phosphate-free artificial tears, punctal plug insertion, reduction of inflammation with topical steroids and cyclosporine A eye drops, and epithelial support with autologous serum eye drops and bandage contact lenses^1,2^. In severe cases, where adequate lubrication of the ocular surface cannot be achieved by conventional means, scleral contact lenses are a viable therapeutic option^5–8^. The lens is filled with liquid saline or eye drops before insertion, creating a fluid reservoir between the corneal surface and the scleral lens. Due to the larger diameter compared to corneal contact lenses, pressure is equally spread over the sclera reducing foreign body sensation and providing a stable and comfortable fit. The lens obviates evaporation and protects the ocular surface from painful epithelial damages and infections due to desiccation^9^. Apart from severe ocular surface disease (e.g. Sjögren’s syndrome, Stevens—Johnson syndrome and ocular GvHD), scleral lenses are used for other ophthalmological diseases such as corneal ectasia, keratoconus and after corneal transplantation as they may increase visual acuity in patients with major refractive errors like severe astigmatism or other corneal irregularities^9^. Adverse events of scleral lenses include trapped air bubbles under the scleral lens, corneal edema, corneal vascularization, papillary conjunctivitis, difficulties with lens insertion or removal, blurriness, discomfort or pain. Although microbial keratitis and corneal ulceration under scleral lens use have been reported in case studies, these adverse events are very rare^9^.

Scleral lenses are fitted individually. Fitting requires different tools of measurement compared to corneal lenses. Anterior segment optical coherence tomography (OCT) has been established in scleral lens fitting to measure the anterior ocular surface and to design an individually fitted scleral lens according to the corneo-scleral transition zone of each patient’s eye.

Over the last years, classification and nomenclature of contact lenses was a subject of debate and different classifications have been suggested. Traditional classification was based on the lens diameter: corneo-scleral (12.9–13.5 mm), semi-scleral (13.6–14.9 mm), mini-scleral (15.0–18.0 mm) and full-scleral (18.1–24.0 mm)^10^. In 2013, the Scleral Lens Education Society suggested a new nomenclature according to size and fit of the lenses^11^. They differentiated between corneal lenses, which rest entirely on the cornea, corneo-scleral lenses which rest partly on the cornea and partly on the sclera and scleral lenses which rest entirely on the sclera. They further divided the scleral lenses into mini-scleral lenses with a size up to 6 mm larger than the horizontally visible iris diameter (HVID) and large-scleral lenses exceeding the HVID by more than 6 mm^11^. Most recently, it was proposed that the terminus “scleral lens” applies to any contact lens vaulting the cornea and limbal area and landing on the conjunctiva^12,13^.

To date, most studies investigating mini-scleral lenses focused on visual rehabilitation in keratoconus and there are no comprehensive studies on their use in ocular GvHD. The aim of this study was to evaluate the effect of mini-scleral contact lenses on visual acuity, eye-related quality of life and the ocular surface in patients with chronic ocular GvHD.

## Methods

This retrospective study was approved and the need for informed consent was waived by the Ethics Committee of the Albert-Ludwigs University of Freiburg (vote n. 131/15). All methods were performed in accordance with the relevant guidelines and regulations. All patients were recruited from the outpatient department of the Eye Center of the University Hospital of Freiburg and were treated for severe dry eye disease due to chronic ocular GvHD. Median Schirmer’s test value before mini-scleral lens fitting was 0 mm (n = 40 eyes, 1st 0/3rd 0,25) and median fluoresceine breakup time (BUT) was 3 s (n = 26 eyes, 1st 1/3rd 6,75). 48% of eyes (30/62) displayed scarring of the tarsal conjunctiva and symblephara were present in 16% (10/62) of eyes. The diagnosis of ocular GvHD was established by a specialist for cornea and external disease in accordance with the diagnostic criteria defined by the German-Austrian-Swiss Consensus Group^14^. Before the indication for mini-scleral contact lenses was made, all patients were started on a conventional treatment regimen including lubricating eye drops and topical ciclosporin A. Punctal occlusion with punctum plugs and/or autologous serum eye drops were incorporated in most patients. Patients who continued to present with severe dry eye symptoms and/or persisting ocular surface damage despite maximal tolerable conventional treatment for ocular GvHD were considered for mini-scleral lens fitting. According to the NIH Consensus Development Project on Criteria for Clinical Trials in Graft-versus-Host Disease, the need for special eye wear results in an organ severity score of 3, the highest obtainable score for the eyes.

For mini-scleral lens fitting, the following parameters were measured using anterior segment OCT (Tomey CASIA©, TOMEY GmbH, Nürnberg, Germany) and corneal topography (Oculus Pentacam©, OCULUS Optikgeräte GmbH, Wetzlar, Germany): radius of curvature, asphericity of the cornea and shape of the anterior sclera. Corneal vaulting and the scleral landing zone were confirmed by fluorescein stain of a test kit lens and the final lens at the slit-lamp.

All patients received individually fitted contact lenses with a maximal diameter of 16.5 mm which, according to the historical classification based on lens diameter, qualifies them as mini-scleral lenses. Each lens was fitted according to the architecture of the individual eye. Sagittal height was chosen depending on the corneal curvature. Thickness of tear film underneath the mini-scleral lens ranged from 0.05 mm at the periphery to 0.2 mm in the center. Mini-scleral lenses consisted of a highly gas permeable material composed of a siloxanyl fluoromethacrylate copolymer (Boston® XO2, specific weight 1.19, DK:L, ISO141).

After manufacturing, all patients were instructed in inserting and removing the lenses. Before insertion, lubricating eye drops without preservatives or sterile saline were filled in the lens cavity. For insertion, patients used fingers or a special contact lens applicator. Patients had to look down while inserting the lens. During the fitting process, sterile, preservative free fluorescein was added to the lens in order to detect corneal damage and to evaluate the tear film reservoir.

Patients were followed up at regular intervals after mini-scleral lens fitting. The frequency of evaluations varied depending on the individual patient’s needs and ranged from monthly to quarterly visits. Visual acuity, OSDI values and corneal fluoresceine staining were chosen as endpoints. At each visit Snellen visual acuity under full optical correction (with spectacles or objective refraction) with and without mini-scleral lenses was assessed. Likewise, patients completed the Ocular Surface Disease Index (OSDI) questionnaire before and after mini-scleral lens wear. Corneal staining was assessed according to the Oxford Grading Scale at baseline and each visit^15^.

Statistical analyses were performed using the R-Platform^16^. Visual acuity was log-transformed before statistical analysis. A paired t-test was used for inference analyses. Summary statistics of continuous data are presented as median and quartiles.

## Results

We included 62 eyes of 31 patients with chronic ocular GvHD. The cohort comprised 17 male and 14 female patients. Median age was 54 years (range 22 to 76 years, 1st 44.5/3rd 62).

Underlying oncologic diseases were acute myeloid leukemia (n = 9), myelodysplastic syndrome MDS (n = 4), chronic myeloid leukemia CML (n = 5), multiple myeloma MM (n = 2), acute lymphoblastic leukemia ALL (n = 4) and others (n = 7). Median time between hematopoietic stem cell transplantation and fitting of mini-scleral lenses was 1960 days (1st 896/3rd 4502).

**Fig. 1:**
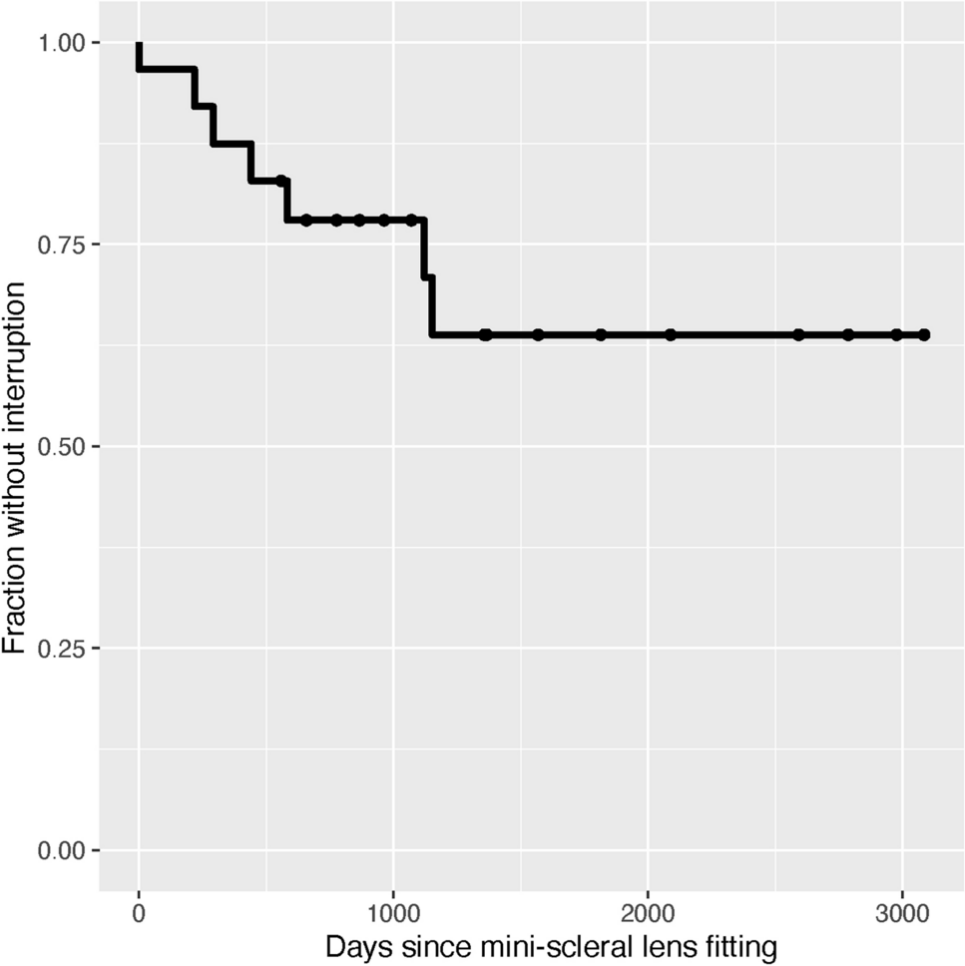
Kaplan–Meier survival analysis shows the fraction of patients without therapy interruption over time. Seven patients aborted mini-scleral lens wear. Most therapy discontinuations occurred during the first 2 years of follow-up.

Discontinuation of mini-scleral lenses was observed in 7 patients (22.6%). Most of these patients discontinued mini-scleral lens wear during the first two years after fitting (Fig. 1). Reasons for discontinuation included difficulties with handling (n = 2), no subjective benefit (n = 2) and recurring herpes simplex virus keratitis (n = 1). For two patients, the reasons for therapy discontinuation were unknown. Median time of follow-up was 717.5 days. There were no adverse events recorded that could be directly attributed to mini-scleral lens wear.

**Fig. 2:**
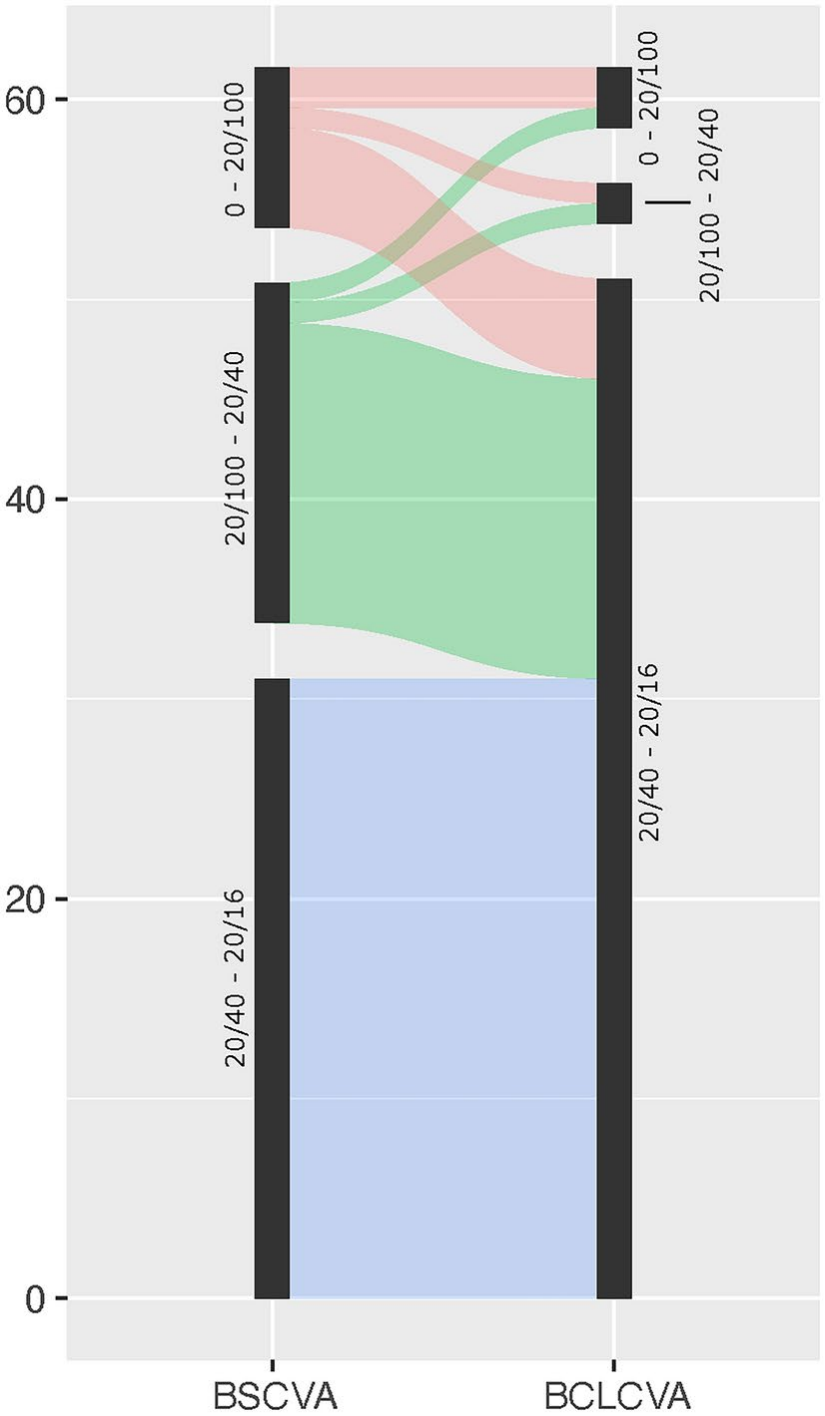
The migration plot shows the changes of visual acuity before mini-scleral lens fitting (best spectacle-corrected visual acuity, BSCVA) and after mini-scleral lens fitting (best contact lens-corrected visual acuity, BCLCVA). Especially eyes with low vision at baseline profited from lens fitting.

Most patients showed an increase in visual acuity with mini-scleral lenses at their last available follow-up visit (Fig. 2). Median Snellen fraction improved to 20/25 (1st 20/30/3rd 20/20) with mini-scleral lenses compared to 20/40 median Snellen fraction with spectacles (1st 20/60/3rd 20/25). Median visual acuity gain was 2.5 EDTRS lines. Especially eyes that exhibited reduced visual acuity at baseline profited from mini-scleral lens use.

**Fig. 3:**
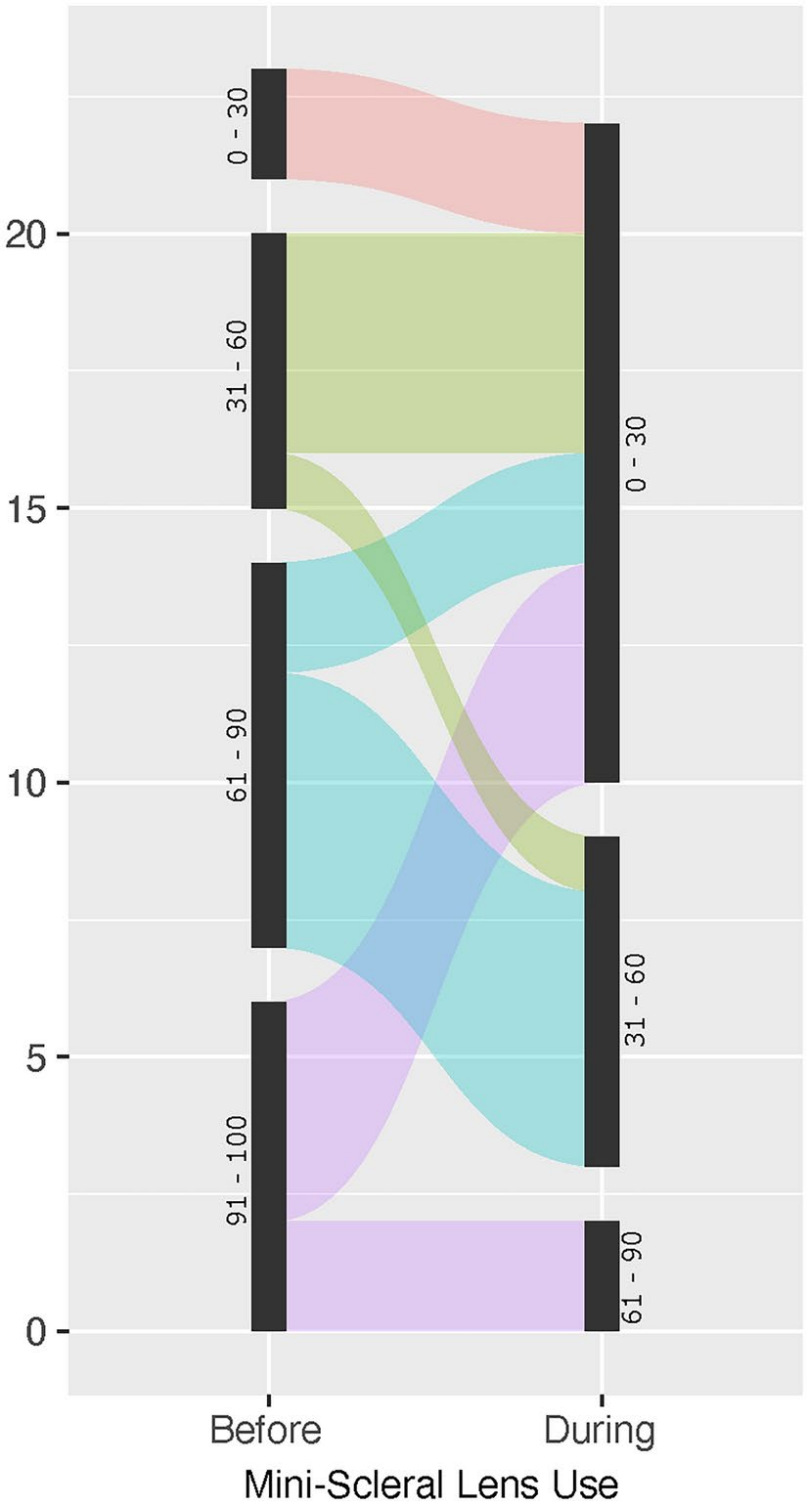
The migration plot shows changes in OSDI scores from before to during mini-scleral lens wear. Most patients reported an improvement of symptoms after lens fitting.

Self-reported eye-related quality of life, assessed by the OSDI questionnaire, improved in almost all patients (Fig. 3). Median OSDI scores improved from 73 before mini-scleral lens fitting to 27 with mini-scleral lens wear. Patients with both severely as well as moderately impaired eye-related quality of life profited similarly.

**Fig. 4:**
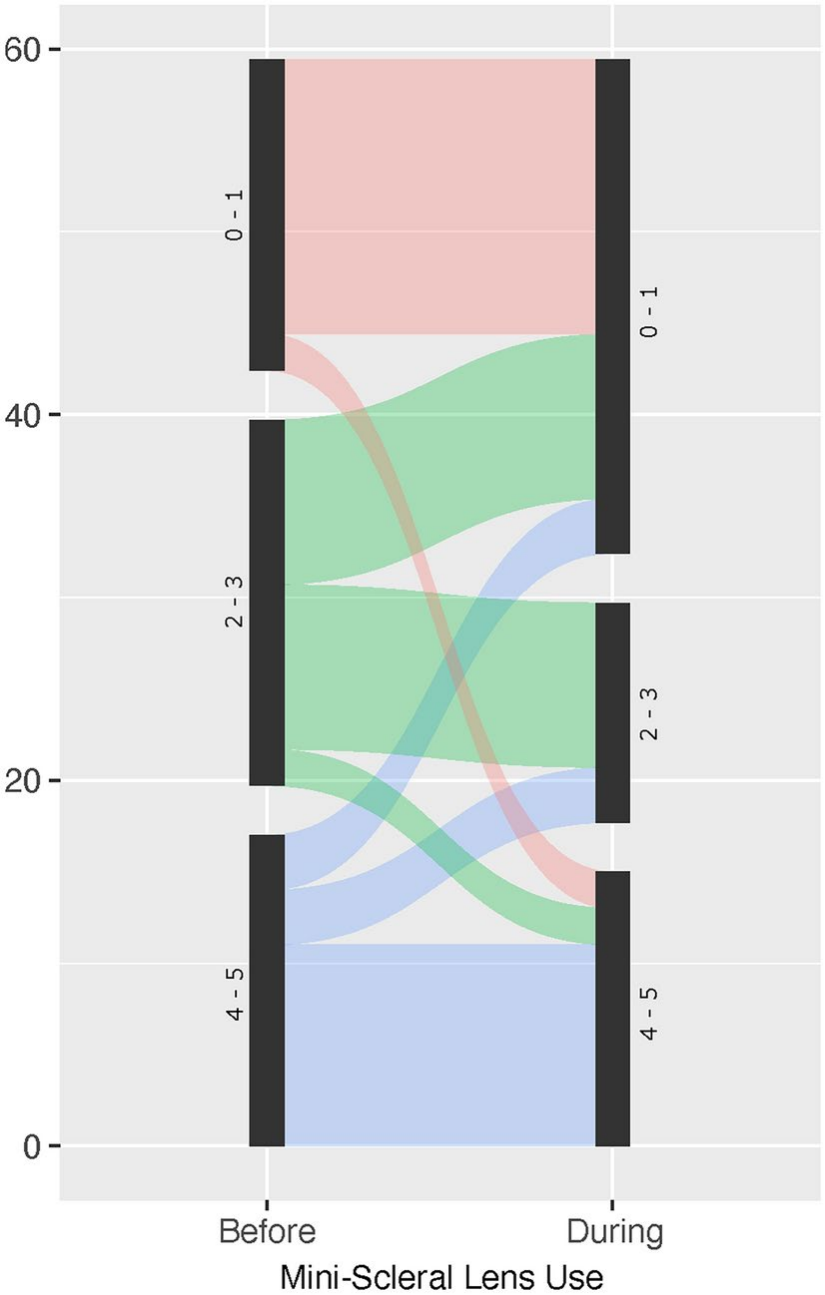
Changes in corneal staining (Oxford grade) after mini-scleral lens fitting. Eyes with more severe corneal staining profited less from mini-scleral lens wear.

The extent of corneal fluorescein staining improved in most patients. Median Oxford grade decreased from 3 (1st 1.0/3rd 4.75) before mini-scleral lens fitting to 1 (1st 0.0/3rd 4.0) with mini-scleral lenses (Fig. 4).

## Discussion

Scleral contact lenses are successfully used in a variety of eye diseases. Two major indications are higher refractive errors, e.g. in keratoconus or after keratoplasty, and severe ocular surface disease^9^. This study analyzes mini-scleral lens wear in patients with ocular GvHD which can be seen as an umbrella term of distinct pathologies of the conjunctiva (e.g. cicatricial conjunctivitis), the cornea (e.g. punctate epithelial erosions) and the ocular adnexa (e.g. Meibomian gland dysfunction) that all contribute to potentially vision threatening ocular surface disease^2^. In principle, mini-scleral or scleral contact lenses may be incorporated in the therapy of severe dry eye disease due to chronic ocular GvHD at any point. However, they are usually used when conventional treatment consisting of intense lubrication, punctal occlusion and topical immunosuppressive therapy fails to adequately relieve patients of dry eye symptoms or when ocular surface damage fails to resolve under maximal tolerable conventional therapy. The successful use of scleral lenses of any size requires the patient’s compliance, as inserting and removing the lens must be practised and may be difficult especially for older patients with comorbidities. In ocular GvHD, fitting a scleral lens can be particularly difficult as conjunctival scarring and the presence of symblephara may significantly reduce the available space for the scleral landing zone.

Previously, other studies showed a beneficial effect of large scleral lenses in ocular surface disease of variable origin. Schornack et al. found an increased visual acuity for neurotrophic and exposure keratopathy, limbal stem cell deficiency and chronic ocular GvHD^7^. Pullum et al. reported their cohort of patients that were treated with scleral lenses for a variety of indications and analyzed visual acuity and ocular comfort. 78.7% of these patients achieved a visual acuity of 20/30 or better. Interestingly, in patients with severe ocular surface disease visual acuity gain was poorer. About 30% of these patients reached a visual acuity above 20/30 but patients mostly benefitted from less pain and reduced discomfort, which is a primary goal in patients with severe dry eye^17^. In a prospective study, the Boston Foundation for Sight found a visual gain by 2 lines or more in 19% for the ocular surface disease group compared to 55% in the ectasia/astigmatism group^18^.

In our study, we exclusively included patients with chronic ocular GvHD. All patients suffered from severe dry eye disease with corneal and conjunctival staining, epithelial damages and tarsal scarring. Few patients presented with symblephara. All patients had primarily received conservative therapy including lubricating eye drops, topical cyclosporine A, steroid or autologous serum eye drops. If severe dry eye persisted, mini-scleral lens fitting was recommended.

Almost all patients included reported an improvement of eye-related quality of life assessed by the OSDI questionnaire. Likewise, median Oxford Grade decreased significantly after mini-scleral lens fitting. This demonstrates that mini-scleral lenses can considerably improve ocular surface alteration in severe dry eye related to ocular GvHD. As expected, patients benefitted from a highly significant increase in visual acuity after mini-scleral lens fitting.

Few studies investigated the use of standard size scleral lenses (diameter greater than 18.0 mm) in chronic ocular GvHD in particular. Schornack et al. found an increased visual acuity in 7 of 10 eyes^19^. Takahide et al. presented an average reduction of OSDI score of 69 points in their survey of 9 GvHD patients^20^. However, those studies included only a small number of patients with chronic GvHD. Theophanous et al. examined 79 eyes of 40 GvHD patients before and after treatment with prosthetic replacement of the ocular surface ecosystem PROSE scleral lenses (Needham, MA, USA)^8^. The authors found that patients who had been treated with PROSE showed significantly reduced morbidity with an average OSDI score reduction of 52 points, a decline in corneal staining and a clinically significant improvement of visual acuity^8^. Another large study on this topic was conducted by Magro and co-workers: Their retrospective multi-center study comprised 60 GvHD patients that were treated with scleral lenses of various diameters^6^. They reported a significant improvement of quality of life, Oxford scores and visual acuity in nearly all patients with good tolerability^6^. All of these studies demonstrate that patients with ocular GvHD significantly benefit from standard size scleral lens wear regarding ocular surface protection, relieve of dry eye symptoms and gain of visual acuity.

In our study, we explicitly investigated the effect of mini-scleral lenses in patients with chronic ocular GvHD. We assumed that mini-scleral lenses provide advantages compared to standard size scleral lenses since they are easier to insert and remove. We used mini-scleral lenses with a maximal diameter of 16.5 mm. They still vault entirely over the cornea, which is important for a stable fit, adequate visual acuity and protection of limbal stem cells. Additionally, corneal hypoxia and associated neo-vascularization may be reduced due to thinner material with better oxygen permeability due to the smaller diameter. Most of our patients profited significantly from mini-scleral lens therapy by improved visual acuity, decreased corneal staining and reduced dry eye symptoms. Interestingly, patients with more severe corneal staining profited less from mini-scleral lenses. Possibly, more advanced ocular surface disease with severe damage to the ocular surface limits the potential for improvement even with mini-scleral lenses. Overall, these results are in line with published literature on the effects of larger scleral lenses in ocular GvHD. In our study however, the rate of patients who were not able to continue therapy (22.6%) with mini-scleral lenses at some point was considerably higher than in studies that investigated standard size scleral lenses. Theophanous et al. reported that 3 out of 40 patients with chronic ocular GvHD stopped scleral lens treatment due to difficulty with application and removal with an average follow up period of 12.3 months^8^. Four patients needed help of a caretaker to handle the scleral lenses. Magro et al. reported therapy discontinuation in 8% of all patients with a median follow up period of 20.5 months^6^. Compared to the cohort reported by Magro et al., our patients exhibited moderately less impaired quality of life as per OSDI scores at baseline. Patients with more severely impaired eye-related quality of life may exhibit a better therapy adherence. Out of all studies, ours provides the longest follow-up time which could explain the higher rates of therapy discontinuation. However, most patients who stopped wearing mini-scleral lenses did so during the first two years after fitting.

To our knowledge, this study is the first one to explore the clinical benefits of mini-scleral lenses in ocular GvHD. It ranges among the largest studies regarding scleral lens wear in ocular GvHD in general and provides a long median follow-up period. Patients with chronic ocular GvHD benefit significantly from mini-scleral lenses in terms of improved vision, decreased corneal staining and increased eye-related quality of life. Therapy discontinuation must be expected in some patients despite clinical improvement and therapy adherence might be improved by careful patient selection before lens fitting. Overall, the improvement of eye-related quality of life, visual acuity and corneal surface disease warrants a broad use of mini-scleral lenses in patients with severe chronic ocular GvHD.

## Limitations of this study

This study has certain limitations, partly due to its retrospective design. Some patients did not present for follow-up after successful lens fitting, did not receive all examinations or did not complete the OSDI questionnaire at follow-up. Reasons for patients becoming lost to follow-up were mostly long distances between their home and the hospital. At least one patient deceased during follow-up. The death was unrelated to contact lens wear. Varying follow-up times may have introduced selection bias, thus affecting the consistency of the results. To address this issue for the endpoint of therapy discontinuation, we used a Kaplan–Meier survival analysis, which is generally considered robust to varying follow-up times. However, an effect on the other endpoints cannot be ruled out. Since both eyes of individual patients were included in the analysis, inter-eye correlations may also have introduced bias. To address this, we decided against the use of inferential statistics. Still, this remains a potential source of bias.

## Supplementary Information

Supplementary Information.

## Data Availability

Data is available on reasonable request from the corresponding author (P.K.).
