# Supplementary material for: Visual and ocular surface benefits of mini-scleral contact lenses in patients with chronic ocular graft-versus-host disease (GvHD)
Source: Sci Rep. 2024 Oct 24;14:25254. doi: 10.1038/s41598-024-76249-5 (PMC11502835; doi:10.1038/s41598-024-76249-5)
Supplement: Supplementary file 1 — Supplementary Information. [file 41598_2024_76249_MOESM1_ESM.docx]

**Supplementary Information for**

**Visual and ocular surface benefits of mini-scleral contact lenses in patients with chronic ocular Graft-Versus-Host Disease (GvHD)**

Philip Keye^1#^, Susanne Issleib^2^, Yvonne Gier^1,3^, Mateusz Glegola^1^, Philip Maier^1^, Daniel Böhringer^1^, Philipp Eberwein^4,1^, Thomas Reinhard^1^

| **Parameters** |  |
| --- | --- |
| Schirmer’s test (n = 40 eyes) | Median 0 mm (1st 0 mm / 3rd 0,25 mm) |
| Breakup time (n = 26 eyes) | Median 3 s (1st 1 s / 3rd 6,75 s) |
| Tarsal scarring (n = 58 eyes) | 48 % |
| Symblephara (n = 58 eyes) | 16 % |

**Table 1.** The table summarizes non-endpoint ocular surface characteristics before mini-scleral lens fitting. Schirmer’s test values were available for 40 of 62 eyes, fluoresceine breakup time was available for 26 of 62 eyes. The presence/absence of tarsal scarring and symblephara could be determined for 58 of 62 eyes.
